# The low‐harm score for predicting mortality in patients diagnosed with COVID‐19: A multicentric validation study

**DOI:** 10.1002/emp2.12259

**Published:** 2020-10-15

**Authors:** Adrian Soto‐Mota, Braulio A. Marfil‐Garza, Erick Martínez Rodríguez, José Omar Barreto Rodríguez, Alicia Estela López Romo, Paolo Alberti Minutti, Juan Vicente Alejandre Loya, Félix Emmanuel Pérez Talavera, Freddy José Ávila Cervera, Adriana Velazquez Burciaga, Oscar Morado Aramburo, Luis Alberto Piña Olguín, Adrian Soto‐Rodríguez, Andrés Castañeda Prado, Patricio Santillán Doherty, Juan O Galindo, Luis Alberto Guízar García, Daniel Hernández Gordillo, Juan Gutiérrez Mejía

**Affiliations:** ^1^ Department of Phyisiology, Anatomy and Genetics The University of Oxford Oxford Oxfordshire United Kingdom; ^2^ Instituto Nacional de Ciencias Médicas y Nutrición Salvador Zubirán Mexico City Mexico; ^3^ Instituto Nacional de Enfermedades Respiratorias Mexico City Mexico; ^4^ Sistema de Salud Christus Muguerza Monterrey Mexico; ^5^ Centro Médico Nacional Siglo XXI Mexico City Mexico; ^6^ Centro Médico Nacional Occidente Guadalajara Mexico; ^7^ Hospital Regional de Alta Especialidad de la Península de Yucatán Mérida Mexico; ^8^ Universidad Anáhuac Mexico City Mexico; ^9^ Hospital de la Beneficencia Española San Luis Potosí Mexico; ^10^ Hospital Regional de Alta Especialidad del Bajío León Mexico; ^11^ Universidad del Valle de México Mexico City Mexico; ^12^ Centro de Investigación en Políticas Población y Salud Mexico City Mexico

**Keywords:** COVID‐19, mortality, prediction, SARS‐COV‐2, score, survival

## Abstract

**Objective:**

We sought to determine the accuracy of the LOW‐HARM score (Lymphopenia, Oxygen saturation, White blood cells, Hypertension, Age, Renal injury, and Myocardial injury) for predicting death from coronavirus disease 2019) COVID‐19.

**Methods:**

We derived the score as a concatenated Fagan's nomogram for Bayes theorem using data from published cohorts of patients with COVID‐19. We validated the score on 400 consecutive COVID‐19 hospital admissions (200 deaths and 200 survivors) from 12 hospitals in Mexico. We determined the sensitivity, specificity, and predictive values of LOW‐HARM for predicting hospital death.

**Results:**

LOW‐HARM scores and their distributions were significantly lower in patients who were discharged compared to those who died during their hospitalization 5 (SD: 14) versus 70 (SD: 28). The overall area under the curve for the LOW‐HARM score was 0.96, (95% confidence interval: 0.94–0.98). A cutoff > 65 points had a specificity of 97.5% and a positive predictive value of 96%.

**Conclusions:**

The LOW‐HARM score measured at hospital admission is highly specific and clinically useful for predicting mortality in patients with COVID‐19.

## 1 INTRODUCTION

### 1.1 Background

Multiple prognostic factors for disease severity in patients diagnosed with coronavirus disease 2019 (COVID‐19) have been identified.^1^, ^2^, ^3^ In this regard, many prognostic scores have already been put forward to predict the risk of death and other outcomes (eg, CALL score, ABC GOALS, Neutrophil‐Lymphocyte index, etc).^4^, ^5^, ^6^ However, hospitals in developing countries often cannot measure some of the variables included in these scores (D‐dimer, ferritin, computed tomography [CT] scans, etc). Moreover, implementation of many of these scores is hampered by the inclusion of subjective variables such as breathlessness,^5^ data on preexisting comorbidities^7^ (making it impossible to reassess prognosis according to the patients’ clinical evolution) or rely on cutoff values that are infrequently met by patients with COVID‐19 in real‐world settings.

### 1.2 Importance

Developing countries have a lower number of critical‐care beds^8^ and specialists per 100,000 people.^9^ Thus, estimating mortality is essential for optimal resource allocation. Prediction tools also have ethical applications and implications. Some triage systems repurpose scores to predict mortality in critical care patients, such as the SOFA (Sequential Organ Failure Assessment) score, as part of their decision framework.^10^, ^11^ However, there is compelling evidence highlighting the importance of generating and using disease‐specific prediction tools or models in pandemic contexts.^12^

Mathematical models for estimating new cases of COVID‐19 in the post‐pandemic period agree there will be >1 “wave” of infections,^13^ and serological surveys for estimating the dynamics of a population's susceptibility, level of exposure, and immunity to the virus support these predictions.^14^, ^15^, ^16^ Therefore, an effective prognostic tool is still relevant even if most countries are already flattening their daily curve of confirmed cases.^17^

Furthermore, having context‐specific predictive accuracy is essential for assisting the decisionmaking process in these extraordinary situations, for objectively tracking clinical status, and for providing realistic and accurate information to patients and their families about prognosis.

### 1.3 Goals of this investigation

This work evaluated the predictive performance of the novel LOW‐HARM score (Lymphopenia, Oxygen saturation, White blood cells, Hypertension, Age, Renal injury, and Myocardial injury) for predicting mortality in patients diagnosed with COVID‐19.

## 2 METHODS

### 2.1 Study design and setting

This work was an observational analytic cohort study. The project and analysis strategy were preregistered at the Open Science Framework (Code: 68er7) on April 21, 2020. The original acronym for this score (HOT CALL) was changed to avoid confusion with the preexisting CALL score.^4^

This study was approved by the Ethics Committee of the Instituto Nacional de Ciencias Médicas y Nutrición on April 29, 2020 (Reg. No. DMC‐3369‐20‐20‐1).

### 2.2 Setting

Data were collected retrospectively (between April 30 and May 20) from the medical records of 12 tertiary care reference hospitals in Mexico. Laboratory results and SpO_2_ values were measured at admission.

The participating institutions and their characteristics were:

1. Instituto Nacional de Ciencias Médicas y Nutrición Salvador Zubirán: Public hospital in Mexico City with 250 beds for COVID‐19 patients.
2. Instituto Nacional de Enfermedades Respiratorias: Public hospital in Mexico City with 150 beds for COVID‐19 patients.
3. Centro Médico Nacional Siglo XXI: Public hospital in Mexico City with 95 beds for COVID‐19 patients.
4. Centro Médico Nacional Occidente: Public hospital in Guadalajara with 100 beds for COVID‐19 patients.
5. Hospital Regional de Alta Especialidad de la Península de Yucatán: Public hospital in Mérida with 124 beds for COVID‐19 patients.
6. Hospital Regional de Alta Especialidad del Bajío: Public hospital in Leon Guanajuato with 40 beds for COVID‐19 patients.
7. Hospital de la Beneficencia Española en San Luis Potosí: Private hospital in San Luis Potosí with 27 beds for COVID‐19 patients.
8. Sistema de Salud: Christus Muguerza: Private hospitals in Monterrey, Puebla, Saltillo, Chihuahua, and Mérida with 142 beds for COVID‐19 patients in total.

### 2.3 Selection of participants

We collected and analyzed data from all patients with severe acute respiratory syndrome coronavirus 2 (SARS‐CoV‐2) infection confirmed by RT‐PCR that were consecutively hospitalized at the already‐mentioned institutions. We excluded from the analysis all patients without a documented clinical outcome (eg, still hospitalized at the moment of data collection, transferred to another hospital, voluntary discharge) or without complete data.

### 2.4 Derivation of the LOW‐HARM Score

The score was constructed based on Fagan's nomogram for Bayes theorem and works as a sequential risk estimation that is modified by the risk factors present in a patient.^18^

The pretest probability of death was obtained using the reported prevalence of death by age group.^19^ After thoroughly reviewing the available literature regarding prognostic factors for mortality in patients hospitalized with COVID‐19, we selected studies reporting enough data to calculate likelihood ratios (LR).^1^, ^2^, ^3^ Afterwards, we chose clinical variables that fulfilled the following criteria:

1. Were linked to or were indicative of end‐organ damage.
2. Had independent pathophysiology (to adhere to the assumptions of Bayesian analysis of independent probabilities).^20^
3. That were routinely available to most hospitals in Mexico (many hospitals do not have access to some follow‐up biomarkers shown to be clinically useful in patients diagnosed with COVID‐19^21^ such as D‐dimer, C‐reactive protein, interleukin‐6 [IL‐6], or ferritin).

We dichotomized the selected variables selected for the model: we defined previous diagnosis of hypertension as present or absent; peripheral capillary oxygen saturation (SpO_2_) as low < 88% and normal > = 88%; cardiac injury as a troponin value > 99th percentile (set by the hospital laboratory), or > 185 U/L for creatine phosphokinase (CPK), or >100 ng/mL for myoglobin, according to Zhou et al^2^; lymphopenia was defined as <800 cells/μL (<0.8 cells/mm3)^2^; kidney injury was defined as a serum creatinine value >1.5 mg/dL; and leukocytosis as a total count >10,000 cells/μL.

We determined the pretest odds (odds of death by age group) using the following formula (pretest odds = pretest probability/(1‐ pretest probability)). For this, we used the reported probability of dying by different age group^19^ as <40 years old = 0.002, 40 to 49 years old = 0.004, 50 to 59 years old = 0.013, 60 to 69 years old = 0. 037, 70 to 79 years old = 0.087, >80 = 0.174.

**The Bottom Line**

The early prediction of poor COVID‐19 outcomes or death could be useful for advancing care. The authors propose the LOW‐HARM score (Lymphopenia, Oxygen saturation, White blood cells, Hypertension, Age, Renal injury, and Myocardial injury) for predicting death after COVID‐19. In a validation using 400 hospitalized patients, the score demonstrated strong discrimination. The LOW‐HARM score could be useful for COVID‐19 prognostication.

LRs for each risk factor were calculated as positive likelihood ratio (+LRs) = sensitivity/(1 − specificity). The calculated +LRs were oxygen saturation <88% = 6.85, previous diagnosis of hypertension = 2.06, elevated troponin, myoglobin, or CPK = 6 (by convergence of their specific LRs from different studies^1^, ^2^, ^3^), leukocyte counts > 10 000 cells/μL = 4.23, lymphocyte counts <800 cells/μL (<0.8 cells/mm3) = 2.89, serum creatinine > 1.5 mg/dL = 4.23.^2^ If any of these findings was absent, the +LR was considered as equal to 1.

With these data, the calculation for the LOW‐HARM score is structured as follows (example in Appendix 1):

1. Pretest odds = pretest probability/(1‐ pretest probability).
2. Posttest odds = (pretest odds) × (LR low SpO2) × (LR previous diagnosis of hypertension) × (LR cardiac injury) × (LR white blood cell count > 10 000 cells/μL) × (LR total lymphocyte count < 800 cells/μL) × (LR acute kidney injury > 1.5 mg/dL).
3. Posttest probability = Posttest odds/(1 + Posttest odds).

### 2.5 Outcomes

The primary outcome was death during hospitalization.

### 2.6 Analysis

Frequency of each risk factor, mean, and standard deviation for the final scores and demographic variables were calculated. Sensitivity, specificity, and predictive values for different score cutoff values were calculated as well. A *P* value of <0.05 for inferring statistical significance was used. Microsoft Excel and STATA v12 software were used for the analysis.

To compare the predictive capacity of our model, we calculated for each case in our sample another 2 recently proposed scores^7^, ^22^ and compared the AUC of their respective receiver operating characteristic (ROC) using DeLong's method^23^ with the STATA function *rroccomp*.^24^

Both scores were chosen because they are also COVID‐19 specific and were also derived from Mexican patients’ datasets. The score from Bello‐Chavolla^7^ was derived using regression models from a publicly available dataset^25^ compiled by the National Institute for Diagnosis and Epidemiological Referral. It assigns points to the identified risk factors and stratifies risk according to their total count. The risk factors it considers are pneumonia (7 points), diabetes and age <40 years (5 points), age >65 years (3 points), chronic kidney disease (3 points), immunosuppression (1 point), chronic obstructive pulmonary disease (1 point), obesity (1 point), diabetes mellitus (1 point), age <40 years (−6 points). The Social Security Mexican Institute (Instituto Mexicano del Seguro Social, IMSS) score can be calculated online^22^ ^;^ it was derived from their institutional epidemiological data and estimates risk from sex, age, weight, hypertension, diabetes, chronic obstructive pulmonary disease, chronic kidney disease, and immunosuppression. However, the methodology and the weight of each risk factor have not been published yet.

### 2.7 Sample size calculation

Mexican official estimations expected at least 10,000, critically ill patients.^26^ To ensure a representative sample, according to the formula for estimating samples from finite populations n = N*X/(X + N – 1), where X = Zα/22 ‐*p*(1‐p)/MOE, Zα/2 is 1.96, MOE is the margin of error, p is 50% (because the actual p is ignored), and N is the population size, data from 385 patients are required to produce a statistically representative sample with an alpha of 0.05%.

## 3 RESULTS

### 3.1 Example of the LOW‐HARM score calculation in a hypothetical case

**FIGURE 1:**
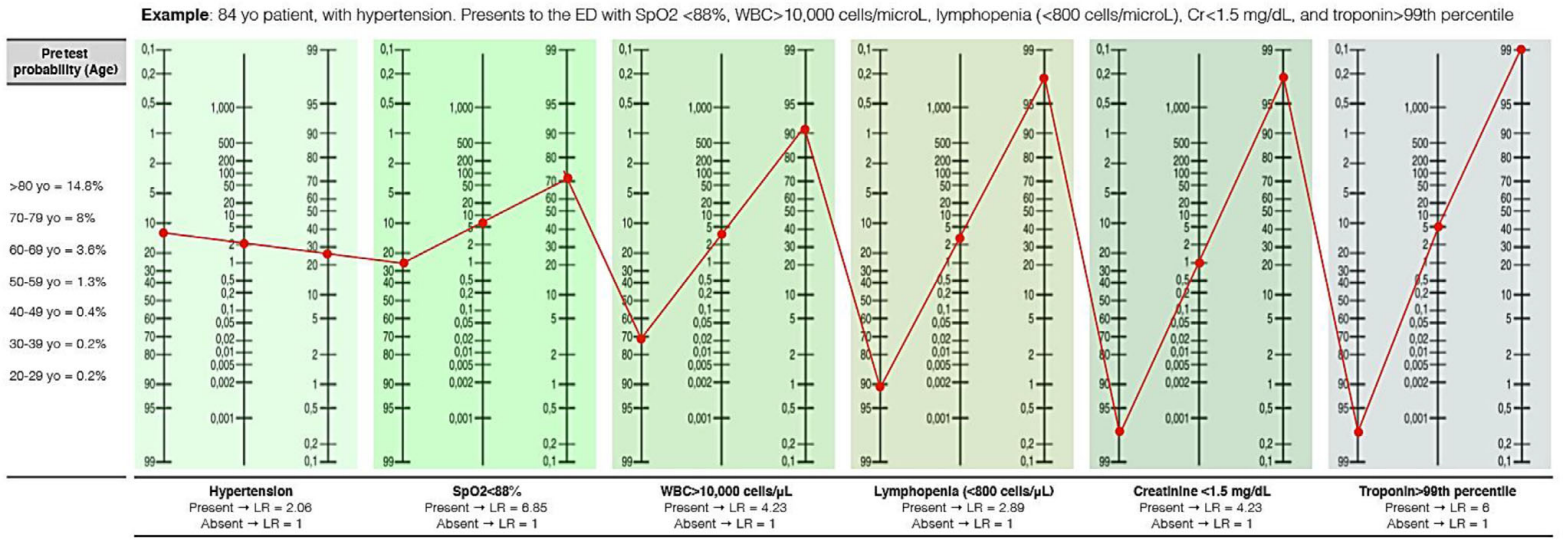
Example of the LOW‐HARM score calculation in a hypothetical case. Based on Fagan's nomogram for Bayes Theorem and using the reported probability of death by age group as the pretest probability. The calculation for the LOW‐HARM score is structured as follows: (1) Pretest odds = pretest probability/(1‐ pretest probability). (2) Post‐test odds = (pretest odds) × (LR SpO2) × (LR diagnosis of Hypertension) × (LR elevation of cardiac enzymes) × (LR white blood cell count > 10 000 cells/mm^3^) × (LR total lymphocyte count < 0.8 cells/ mm^3^) × (LR serum creatinine > 1.5 mg/dL). (3) Posttest probability = Posttest odds/(1 + Post‐test odds). In this hypothetical case, pretest probability starts at 14.8%, is converted to odds and is multiplied by the LR+ of each risk factor when it is present or by 1 when it is absent (in this example, serum creatinine only). Finally, posttest odds are transformed back to posttest probabilities. For ease of use, this process is automated in a freely available web app: lowharmcalc.com. Cr, creatinine; LOW‐HARM, Lymphopenia, Oxygen saturation, White blood cells, Hypertension, Age, Renal injury, and Myocardial injury; LR, likelihood‐ ratios

To illustrate how the LOW‐HARM score is calculated we consider an 83‐year‐old patient with hypertension who has been diagnosed with COVID‐19 and admitted to the hospital (Figure 1). At admission, he presents with a SpO_2_ < 88%, leukocytes >10,000 cells/mm^3^, lymphocytes <0.8 cells/ mm^3^, troponin > 99th percentile, and a serum creatinine < 1.5 mg/dL. Due to his age, this patient's pretest probability of dying is 14.8% (according to Centers for Disease Control and Prevention reports^19^). This probability is converted to pretest odds (pretest odds = pretest probability/(1‐ pretest probability) = 0.174). This value is then multiplied by the calculated LR+ for each risk factor to obtain posttest odds (hypertension = 2.06, SpO2 < 88% = 6.85, elevated troponin = 6, leukocyte count > 10 000 cells/μL = 4.23, lymphocyte count < 800 cells/μL = 2.89, serum creatinine > 1.5 mg/dL = 4.23) or by 1 when any of these is absent (in this case, serum creatinine, which was <1.5 mg/dL). Finally, posttest odds are transformed back to posttest probabilities (posttest probability = posttest odds/(1 + posttest odds)). For this hypothetical patient, the posttest probability of death during his hospitalization is 99% (Figure 1). For ease of use, this process is automated in a freely available web app: www.lowharmcalc.com.

### 3.2 Characteristics of study subjects

We obtained data from 438 patients. A total of 38 patients were excluded, leaving 200 patients per group. Their clinical and demographic characteristics are summarized in Table 1. All components of the LOW‐HARM score were significantly more frequent in the group of patients who died than in the group of patients who survived their hospitalization (Table 2).

**TABLE 1:** Patients’ characteristics

Variable	Survivors (n = 200)	Deaths (n = 200)	*P* value^a^
Sex (%)			
Female	67 (33.5)	53 (26.5)	
Male	133 (66.5)	147 (73.5)	0.12
Age group, years (%)			
20–29	18 (9)	2 (1)	
30–39	40 (20)	14 (7)	
40–49	43 (21.5)	28 (14)	<0.01
50–59	54 (27)	57 (28.5)	
60–69	28 (14)	54 (27)	
70–79	16 (8)	33 (16.5)	
>80	1 (0.5)	12 (6)	
Weight, kg (SD)	80 (15.0)	80.2 (17.2)	0.80
Height, cm (SD)	168 (9)	164.8 (9.2)	<0.01
Body‐mass index (SD)	28 (5)	29.5 (5.8)	0.01
Obesity (BMI ≥ 30 kg/m^2^) (%)	54 (27)	78 (39)	0.01
Required IMV (%)	22 (11)	123 (61.5)	<0.01
Lengthofstay, days (SD)	10 (7)	8.1 (7.3)	<0.01
Diabetes mellitus (%)	46 (23)	78 (36)	<0.01
Pregnancy (%)	2 (1)	0 (0)	0.15
Smoking (%)	24 (12)	25 (12.5)	0.75
Immunocompromised (%)	9 (4.5)	13 (6.5)	0.38
COPD (%)	3 (1.5)	35 (17.5)	<0.01
CKD (%)	5 (2.5)	8 (4)	0.58
CAD (%)	3 (1.5)	8 (4)	0.12

BMI, body mass index; CAD, coronary artery disease; CKD, chronic kidney disease; COPD, chronic obstructive pulmonary disease; IMV, invasive mechanical ventilation; SD, standard deviation.

^a^ Categorical variables were compared using a Xi^2^ test, continuous variables were compared using an unpaired Student *t* test.

**TABLE 2:** Frequency of each LOW‐HARM score component according to clinical outcome

Variable	Survivors (n = 200)	Deaths (n = 200)	*P* value^a^
Lymphocytes < 800 cells/ μL	65 (32.5)	146 (73)	<0.01
SpO_2_ < 88% (%)	73 (36.5)	191 (95.5)	<0.01
White blood cells > 10,000 cells/μL	20 (10)	113 (56.5)	<0.01
Hypertension (%)	40 (20)	95 (47.5)	<0.01
Serum creatinine > 1.5 mg/dL (%)	4 (2)	78 (36)	<0.01
Cardiac injury^b^ (%)	22 (11)	118 (59)	<0.01
*Creatine phosphokinase* *> 185 U/L*	16 (8)	38 (19)	
*hsTpI > 99th P100*	5 (2.5)	78 (39)	
*Myoglobin*	1 (0.5)	2 (1)	

SpO2, peripheral capillary oxygen saturation.

^a^ Categorical variables were compared using a Xi^2^ test.

^b^ Cardiac injury was defined as an elevation of high‐sensitivity troponin I > 99th percentile, creatine phosphokinase serum levels > 185 U/L or serum myoglobin levels > 100 ng/mL.

### 3.3 Predictive performance of the LOW‐HARM score

Sensitivity, specificity, positive and negative predictive values, and their corresponding AUCs for different cutoff values are presented in Table 3. The cutoff value of 25 has the highest AUC (0.9); however, it has a specificity (the probability of correctly identifying a survivor with a score lower than the cutoff value) that is <90%. In contrast, the cutoff value of 65 has a specificity of 97.6% and a positive predictive value of 96% (the probability of death if presenting a score higher than the cutoff value).

**TABLE 3:** Sensitivity, specificity, positive and negative predictive values, and AUCs for different score cutoffs of the LOW‐HARM score

Cutoff	Se,^a^ %	Sp, %	PPV, %	NPV, %	AUC (95% CI)
0	100	0	–	–	–
5	99.5	64	73	99	0.82 (0.79–0.89)
10	99	78.5	82	99	0.89 (0.86–0.92)
15	96	81	83.5	95	0.89 (0.85–0.92)
20	92.5	85	86	92	0.89 (0.86–0.92)
25	91.5	89	90	91	0.90 (0.87–0.93)
30	85.5	89.5	89	86	0.87 (0.84–0.91)
35	82	92	91	84	0.87 (0.84–0.90)
40	82	92	91	84	0.87 (0.84–0.91)
45	77	94	93	80	0.86 (0.82–0.89)
50	75.5	94.5	93	79.5	0.85 (0.82–0.88)
55	69.5	95	93	76	0.82 (0.79–0.86)
60	68.5	95.5	94	75	0.82 (0.78–0.86)
65	63	97.5	96	72.5	0.80 (0.77–0.84)
70	58	98	97	70	0.78 (0.74–0.82)
75	57	98	96.5	69.5	0.78 (0.74–0.81)
80	51	99	98	67	0.75 (0.71–0.79)
85	43.5	99.5	99	64	0.72 (0.68–0.75)
90	35	99.5	99	60.5	0.67 (0.64–0.71)
95	23.5	100	100	56.5	0.62 (0.59–0.65)
100	0	100	–	–	–

AUC, area under the receiver operating characteristic curve; NPV, negative predictive value; PPV, positive predictive value; Se, sensitivity; Sp, specificity.

^a^Positivity defined as having a score above the cutoff value and dying.

**FIGURE 2:**
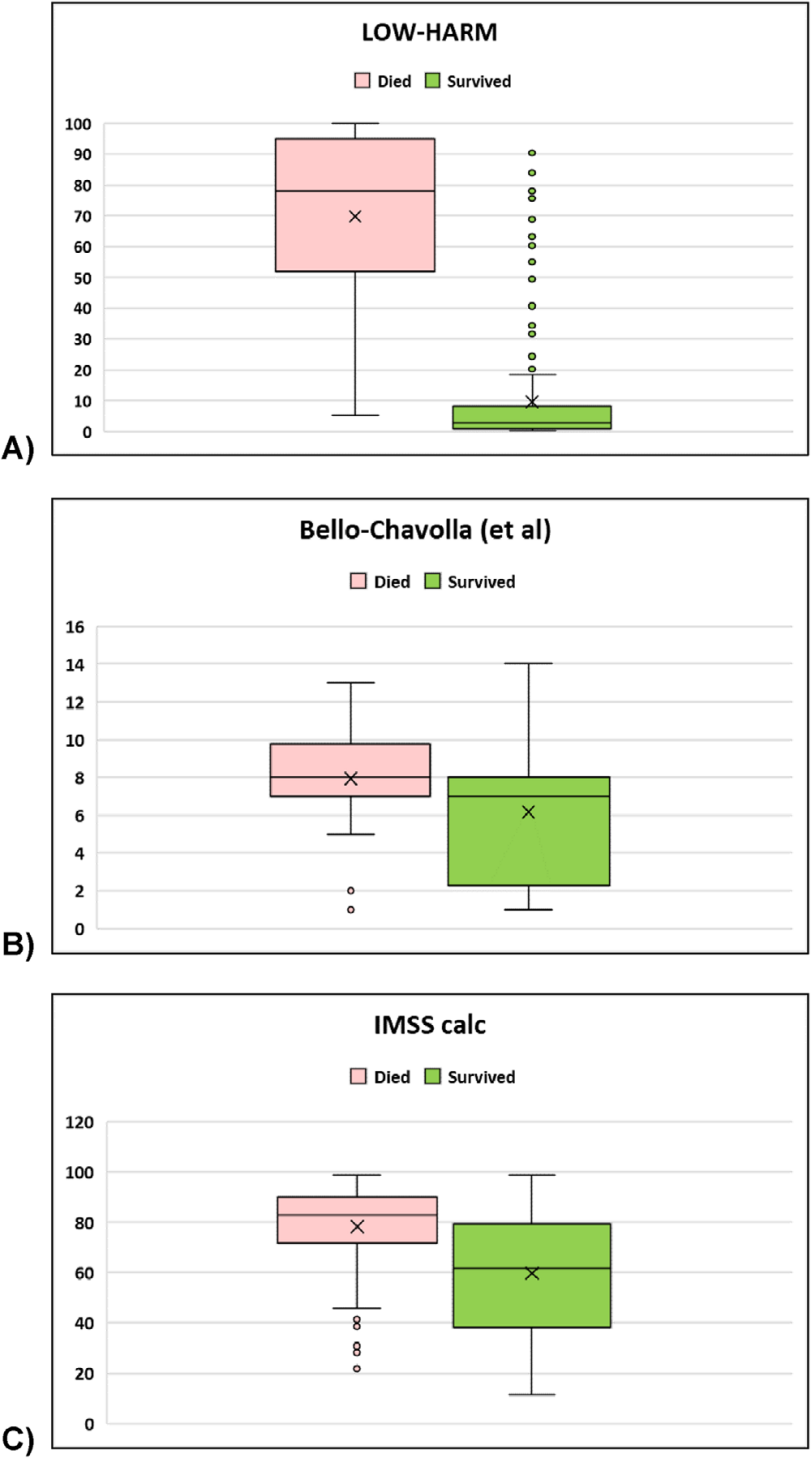
Distribution of scores according to clinical outcome in our sample using different scores. n = 200 per group. For all scores, *P* < 0.01 when compared with Student's *t* test for independent samples. However, the difference between means was larger for the LOW‐HARM score because of better identification of survivors (60 vs 2 vs 18). IMSS, Instituto Mexicano del Seguro Social

**FIGURE 3:**
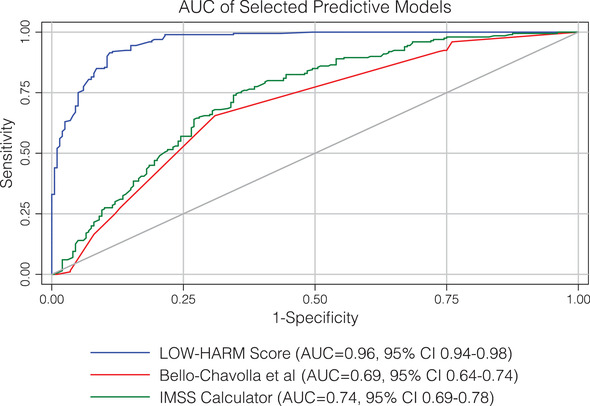
AUC of different mortality scores. AUCs were compared using DeLong's method. AUC, area under the curve ; CI, confidence interval; IMSS, Instituto Mexicano del Seguro Social; LOW‐HARM, Lymphopenia, Oxygen saturation, White blood cells, Hypertension, Age, Renal injury, and Myocardial injury

The mean LOW‐HARM score for deaths was 70 (SD: 28) versus 10 (SD: 17) for survivors (mean: 10, SD: 17), *P* = < 0.01. We calculated other scores proposed for the Mexican population in our sample^7^, ^23^ and, for all scores, the mean scores were significantly different between groups when compared with 2‐tailed Student paired *t* tests. However, the difference between means was larger for the LOW‐HARM score mostly because of a better identification of survivors (60 vs 2 vs 18 points). These findings are illustrated in Figure 2. To further compare the predictive performance of the 3 scores, we compared their AUCs using DeLong's method.^23^ Figure 3 depicts all 3 AUCs and their confidence intervals and shows that the LOW‐HARM score had a greater AUCs, as compared to Bello‐Chavolla et al and the IMSS calculator.^7^, ^22^

## 4 LIMITATIONS

It is possible that the rate of change in the score (showing either improvement or worsening) has more prognostic value. At the same time, it is possible that likelihood ratios for the variables in our score can be refined as studies involving more patients are published. However, these comparisons are outside the reach of this study and are actively being investigated.

Even when we obtained LRs for our score from studies done in populations with different ethnicity, one of the limitations of our study is that Mexico has a high proportion of young people living with hypertension, diabetes, and obesity.^27^, ^28^, ^29^ Therefore, the validity and accuracy of the LOW‐HARM may vary in countries with a different demographic composition or with a different burden of chronic disease. This should be explored further.

Using a score cutoff is as useful as the number of times this cutoff is met. In this case, 105/400 (26%) patients had a score above 65, which means it is possible to predict mortality with a specificity of 97.5% and a positive predictive value of 96% in more than a quarter of hospital admissions. On the other hand, it should be considered that mortality in hospitalized patients with COVID‐19 has markedly improved because of the refinement of triage systems, the standardization of therapeutic protocols and awareness of early symptoms in the general population. Therefore, end‐organ damage at admission is expected to be less frequent.

## 5 DISCUSSION

Accurately predicting which patients will not survive hospitalization can guide optimal resource allocation at emergency departments and support clinicians in their decisionmaking process. Additionally, accurate prediction of certain outcomes can help informing patients and their relatives about prognosis.

We present the LOW‐HARM score, a novel, easy‐to‐use, and easy‐to‐measure tool to predict mortality in hospitalized patients diagnosed with COVID‐19. To our knowledge, this is the first COVID‐19‐specific mortality prediction specifically designed for hospitals without access to the currently advised follow‐up inflammation markers in patients with COVID‐19^21^ (such as C‐reactive protein, D‐dimer, IL‐6, etc). However, it has advantages relevant to more resourceful scenarios as well.

Even in a resourceful environment, many of the variables used in other scores are not dynamic and/or are not measured frequently (comorbidities, CT scans, IL‐6, among others). Contrastingly, cardiac enzymes are repeatedly measured as an independent prognostic tool and complete blood counts and serum creatinine levels are measured almost daily allowing to update predictions. This is a major advantage for the LOW‐HARM score, which allows dynamic reassessment and fine‐tuning of its predictive capacities as the patient's clinical condition evolves during hospitalization. We created a free digital tool where the calculation of the LOW‐HARM score can be automatized, allowing quick, frequent (even daily), and reproducible predictions as the patient's status evolves.^30^

Additionally, simply adding comorbidities does not consider how controlled those diseases were in the first place.^5^, ^7^, ^22^ For example, a 50‐year‐old patient living with diabetes without proper follow‐up or treatment does not have the same prognosis as one who has successfully controlled their disease; similar principles would apply to other comorbidities such as cancer or immunosuppression. Conversely, end‐organ damage is a more effective outcome predictor, for COVID‐19 and, perhaps, for any disease. If prognosis estimations rely only on preexisting comorbidities or fixed data, the prognostic value of assessing the severity of the disease is not pondered. This can yield to inaccurate predictions and/or misclassification, as it was shown in our sample, where many patients who survived obtained a high result in other scores due to their age or previous comorbidities.

Having a cutoff value can be useful for decisionmaking. A frequently used method for choosing a cutoff value is to use the value with the largest AUC. In our score, the largest AUC was observed using a cutoff of 25 (0.90, 95% confidence interval: 0.87–0.93). However, because it is possible that clinicians at emergency departments could use the dichotomized version of the score to allocate healthcare resources, we propose a 65‐point cutoff value because, in this context, we believe it is preferable to choose a cutoff with a high specificity to correctly identify the highest number of patients that will survive, even if they ultimately die (therefore preserving their “eligibility” for resource allocation), than having a high sensitivity and identifying the highest number of patients that will die, even when they could have survived (therefore denying their “eligibility” for resource allocation).

## 6 CONCLUSION

The LOW‐HARM score measured at the time of admission has high accuracy in predicting mortality in patients diagnosed with COVID‐19 requiring hospitalization. This score provides a disease‐specific tool that uses easily obtainable variables making it useful for resource‐limited settings.

## CONFLICT OF INTEREST

None of the authors declares financial interests or personal relationships that could have influenced the work reported in this study.

## AUTHOR CONTRIBUTIONS

ASMdesigned the prediction score. JGM, BAMG, and ASR collaborated in designing and executing data analysis. EMR, JOBR, AELR, PAM, JVAL, FEPT, FJAC, AVB, OMA, LAPO, and ACP obtained clinical and demographic data. PSD, JOG, LAGG, and DHG provided mentorship. All authors revised this manuscript and data analysis.
